# Genomic Selection for Prediction of Fruit-Related Traits in Pepper (*Capsicum* spp.)

**DOI:** 10.3389/fpls.2020.570871

**Published:** 2020-10-28

**Authors:** Ju-Pyo Hong, Nayoung Ro, Hea-Young Lee, Geon Woo Kim, Jin-Kyung Kwon, Eiji Yamamoto, Byoung-Cheorl Kang

**Affiliations:** ^1^ Department of Agriculture, Forestry and Bioresources, Research Institute of Agriculture and Life Sciences, Plant Genomics Breeding Institute, College of Agriculture and Life Sciences, Seoul National University, Seoul, South Korea; ^2^ National Agrobiodiversity Center, National Institute of Agricultural Sciences, Rural Development Administration, Jeonju, South Korea; ^3^ Graduate School of Agriculture, Meiji University, Tokyo, Japan

**Keywords:** pepper, fruit-related traits, core collection, model training, population structure, breeding, genomic selection, cross-validation

## Abstract

Pepper (*Capsicum* spp.) fruit-related traits are critical determinants of quality. These traits are controlled by quantitatively inherited genes for which marker-assisted selection (MAS) has proven insufficiently effective. Here, we evaluated the potential of genomic selection, in which genotype and phenotype data for a training population are used to predict phenotypes of a test population with only genotype data, for predicting fruit-related traits in pepper. We measured five fruit traits (fruit length, fruit shape, fruit width, fruit weight, and pericarp thickness) in 351 accessions from the pepper core collection, including 229 *Capsicum annuum*, 48 *Capsicum baccatum*, 48 *Capsicum chinense*, 25 *Capsicum frutescens*, and 1 *Capsicum chacoense* in 4 years at two different locations and genotyped these accessions using genotyping-by-sequencing. Among the whole core collection, considering its genetic distance and sexual incompatibility, we only included 302 *C. annum* complex (229 *C. annuum*, 48 *C. chinense*, and 25 *C. frutescens*) into further analysis. We used phenotypic and genotypic data to investigate genomic prediction models, marker density, and effects of population structure. Among 10 genomic prediction methods tested, Reproducing Kernel Hilbert Space (RKHS) produced the highest prediction accuracies (measured as correlation between predicted values and observed values) across the traits, with accuracies of 0.75, 0.73, 0.84, 0.83, and 0.82 for fruit length, fruit shape, fruit width, fruit weight, and pericarp thickness, respectively. Overall, prediction accuracies were positively correlated with the number of markers for fruit traits. We tested our genomic selection models in a separate population of recombinant inbred lines derived from two parental lines from the core collection. Despite the large difference in genetic diversity between the training population and the test population, we obtained moderate prediction accuracies of 0.32, 0.34, 0.50, and 0.48 for fruit length, fruit shape, fruit width, and fruit weight, respectively. This use of genomic selection for fruit-related traits demonstrates the potential use of core collections and genomic selection as tools for crop improvement.

## Introduction

Pepper (*Capsicum* spp.) is an important vegetable crop, consumed as a spice and as a fresh vegetable around the world. It is an important source of nutrients such as vitamins C, E, and provitamin A (Palevitch and Craker, 1996). Moreover, pepper extracts such as capsaicin and carotenoids are used for industrial and pharmaceutical purposes. Fruit-related traits of pepper, such as fruit size and pericarp thickness, are critical determinants of quality and are controlled by quantitative trait loci (QTLs). Many QTL analyses and genome-wide association studies (GWASs) of these fruit-related traits have been conducted and reported representative major QTLs for fruit shape like *fs2.1*, *FrSHP2.1*, and *fs3.1* (Chaim et al., 2001; Rao et al., 2003; Zygier et al., 2005; Barchi et al., 2009; Borovsky and Paran, 2011; Mimura et al., 2012; Han et al., 2016; Hill et al., 2017; Chunthawodtiporn et al., 2018; Colonna et al., 2019). However, these studies have focused only on identifying the variants linked to theses quantitative traits but not on applying those variants for variety improvement.

Quantitative traits are difficult to improve through breeding programs since multiple loci with small effects control phenotypic variation of these traits. In genomic selection (GS; Meuwissen et al., 2001), a genomics-based strategy first used in dairy cattle breeding, genome-wide markers are used to predict the phenotypic variation of complex traits. GS is conducted with two populations, a training population with both genotypic and phenotypic information and a test population that has only genotypic data. Statistical models estimate the sum of marker effects from the training population. Estimated marker effects models are used to predict phenotypic values called genomic estimated breeding values (GEBVs). Cross-validation is often then implemented to find the best-fitting model for evaluating the GEBVs in the test population (Desta and Ortiz, 2014) and generate the GS evaluation (Crossa et al., 2011). In cross-validation, the training population is randomly divided into *k* groups, and these groups are assigned to other training sets and validation sets. Training sets are used to estimate marker effects, and GEBVs for the validation sets are calculated using the models. Correlation between predicted GEBVs and phenotypic information of the validation sets indicates the prediction accuracy of the models.

GS has become a promising method in plant breeding as well as in animal breeding. However, GS has been mainly focused on staple crops such as maize (*Zea mays*), wheat (*Triticum aestivum*), barley (*Hordeum vulgare*), and potato (*Solanum tuberosum*). Investigation of GS performance for crop improvement was first conducted in maize (Lorenzana and Bernardo, 2009) followed by barley (Lorenzana and Bernardo, 2009; Crossa et al., 2010) and wheat (Crossa et al., 2010; Heffner et al., 2011). In the Solanaceae family, GS has been performed in tomato and potato. Genomic prediction was evaluated for agronomic traits such as yield, nutritional quality (Habyarimana et al., 2017; Stich and Van Inghelandt, 2018), and resistance to diseases such as late blight (*Phytophthora infestans*) and common scab (*Streptomyces scabies*) in tetraploid potato (Enciso-Rodriguez et al., 2018). Yamamoto et al. (2017) conducted genomic prediction for soluble solid content and general yield in tomato (*Solanum lycopersicum* L.). However, the potential of GS in pepper remains to be demonstrated.

In this study, we tested the potential of GS for the fruit-related traits: fruit length, fruit shape, fruit width, fruit weight, and pericarp thickness in pepper. We cultivated 351 accessions from the pepper core collection (229 *Capsicum annuum*, 48 *Capsicum baccatum*, 48 *Capsicum chinense*, 25 *Capsicum frutescens*, and 1 *Capsicum chacoense*) in 3 years at two different locations. Among whole accessions, considering its crossability, we selected *C. annuum* complex (229 *C. annuum*, 48 *C. chinense*, and 25 *C. frutescens*) for training the models. Through cross-validations, we evaluated the effects of trait architecture and heritability of fruit-related traits, population structure of the training population, and the number of markers on prediction accuracies. Finally, we tested our GS models in a population of recombinant inbred lines (RILs) derived from two parental lines from the core collection.

## Materials and Methods

### Plant Materials

Plant materials were sourced from the previously constructed *Capsicum* core collection (Lee et al., 2016) of the Horticultural Crops Breeding and Genetics Lab (Seoul National University, Korea). The population included five species: 229 *C. annuum*, 48 *C. baccatum*, 48 *C. chinense*, 25 *C. frutescens*, and 1 *C. chacoense*. However, to reduce population structure, *C. baccatum* and *C. chacoense* were excluded in cross-validation and tests of genomic prediction. Plants were grown at RDA-GenBank in Jeonju, Republic of Korea, for measurement in 2015 and 2017. In 2018 and 2019, all plants were grown at Hana Seed Co., Ltd. in Anseong, Republic of Korea. Over 4 years, three plants per accession were randomly planted and cultivated in greenhouses. Three fruits per plant were harvested and evaluated for five fruit traits (fruit length, fruit shape, fruit width, fruit weight, and pericarp thickness).

Pepper seeds from the core collection were sown in early March, and seedlings were transplanted in early May. Since the maturation time of fruits varied among the accessions, fruits were harvested through mid to late August. Five fruit-related traits were measured.

Recombinant inbred lines (RILs) derived from a cross between “Perennial” (P for PD) and “Dempsey” (D for PD) were used as a test population to evaluate genomic prediction models (Han et al., 2016). The PD RIL population comprised 122 lines. Four fruit-related traits were measured. The population was grown at Hana Seed Co., Ltd., in Anseong (2011, 2012a) and at Seoul National University farm in Suwon, Republic of Korea (2012b). All plants were grown in plastic greenhouses at both locations; however, plants in Anseong were grown in soil beds, while plants in Suwon were grown in pots. Five plants were grown for each line in the position of which each line was designated randomly. Among the reported 18 horticultural traits (Han et al., 2016), we utilized four fruit-related traits (fruit length, fruit shape, fruit width, and fruit weight) for testing of genomic prediction.

### Library Construction

Genotyping-by-sequencing was conducted for accessions from the pepper core collection as previously described (Lee et al., 2016). Libraries were generated manually by digestion of gDNA with *Pst*I/*Mse*I and *Eco*RI/*Mse*I. Library adapters were then ligated to digested gDNA, and the libraries were amplified using selective primers containing TA. Constructed libraries were pooled into five tubes and sequenced in separate lanes on a HiSeq 2000 (Illumina, San Diego, CA, USA) at Macrogen (Seoul, Republic of Korea). Whole-genome re-sequencing of the PD RILs population was conducted as previously described (Han et al., 2016).

### Genotype Data Generation and Analysis

Adapter trimming and quality control of raw data were conducted using the CLC Genomics Workbench v6.5 (Qiagen, Aarhus, Denmark) with a minimum read length of 80 bp and minimum quality score of Q20. Filtered raw reads of core collection accessions and PD RILs were aligned against the newly constructed high-quality reference genome of *C. annuum* “Dempsey” (unpublished) using the Burrows-Wheeler Aligner (BWA; Li and Durbin, 2009). Aligned mapping files were sorted and read-grouped using Genome Analysis Toolkit (GATK; DePristo et al., 2011). Sorted mapping files were genotyped together using GATK Haplotype Caller 3.8 to generate variant call format (VCF) files. These VCF files were generated by joint genotyping of 472 samples: 350 from the pepper core collection and 122 PD RILs. Raw genotyped data were filtered using GATK VariantFIltration with the following criteria: MQ < 40.0, SOR > 3.000, QD < 2.00, FS > 60.000, MQRankSum< −12.500, ReadPosRankSum< −8.000. Single-nucleotide polymorphisms (SNPs) with greater than 70% missing markers and minor allele frequency (MAF) less than 0.05 were removed from filtered VCF files using the VCFtools software (Danecek et al., 2011). Missing genotypes of VCF files were imputed and phased using BEAGLE through the R package “synbreed” (Wimmer et al., 2012).

### Population Structure

To validate population structure information for the core collection from a previous study (Lee et al., 2016), principal component analysis (PCA) and hierarchical clustering were performed using new genotypic data for the core collection obtained in this study. PCA of the pepper core collection with genome-wide SNPs was performed using the R package “poppr” (Kamvar et al., 2014).

Genetic clustering analysis was conducted using *ADMIXTURE* v1.3 (Alexander et al., 2009) to estimate the proportion of ancestral information in the pepper core collection. *ADMIXTURE* was run with the number of ancestral populations (*K*) from 1 to 12, and the results were validated with fivefold cross-validation.

Hierarchical clustering was conducted using the unweighted pair group method with arithmetic mean (UPGMA). Genetic distance was estimated based on Euclidean distance using the R package “poppr” (Kamvar et al., 2014). All plots of population structure were generated using the R package “ggplot2” (Wickham, 2016).

### Phenotypic Data Analysis and Heritability

Among the various agronomic traits of pepper, five fruit-related quantitative traits (fruit length, fruit shape, fruit width, fruit weight, and pericarp thickness) were selected for testing traits of genomic prediction. Three plants (biological replications) of each line were planted, and three randomly selected fruits from each plant were measured to generate raw phenotypic data. Fruit length of each fruit was measured by ruler, and fruit width and pericarp thickness were measured by caliper. Each fruit was weighed on a digital weighing scale to measure fruit weight. Fruit shape was defined as the ratio of fruit length to fruit width. To confirm the difference among accessions in the core collection, we conducted pairwise T-test between *C. annuum* and other accessions after analysis of variance (ANOVA). Four fruit-related traits (fruit length, fruit shape, fruit width, and fruit weight) were measured for the PD RILs used as a test population.

In this study, the experimental design was highly imbalanced. To control imbalanced phenotypic data in the core collection, best linear unbiased predictor (BLUP) values for each core collection line (genotype) were calculated using the R package “lme4” (Bates et al., 2015). The random-effects model for fruit-related traits included genotype, year, location, and genotype-environment (G × E) interaction. Variance components were estimated from the random-effects model, and these variance components were used to estimate broad-sense heritability.

### Genomic Prediction Method

Ten different genomic prediction models were used to investigate the best models for fruit-related traits in the pepper core collection: linear methods gblupRR, Ridge regression, LASSO, Elastic net, Bayesian LASSO (BL), extended Bayesian LASSO (EBL), Bayes-B, Bayes-C, and nonlinear methods reproducing kernel Hilbert space (RKHS) and random forest. gblupRR and RKHS are kernel methods; gblupRR estimates the variance of genetic effects of markers based on a single linear kernel, whereas RKHS is based on multiple Gaussian kernels. Each genomic prediction method was implemented by various R packages: “rrBLUP” for gblupRR and RKHS, “glmnet” for Ridge regression, LASSO, and Elastic-Net, “VIGoR” for BL, EBL, Bayes-B, and Bayes-C, and “randomForest” for random forest (Breiman, 2001; Endelman, 2011; Simon et al., 2011; Onogi and Iwata, 2016).

### Evaluation of Genomic Prediction

The accuracy of genomic prediction across the training population was estimated using a 10-fold cross-validation methodology. The whole training population was divided equally into 10 groups. Among the subgroups, nine groups were used as training sets, and one group was randomly assigned as the validation set. This procedure was iterated in 10 different patterns for each trait. The prediction accuracy was recorded by Pearson correlation between predicted values and observed values in every pattern, and the mean of 10 iterations was recorded as the result for the specific trait.

To investigate the effect of marker density, various marker sets were generated. SNPs were pruned from the all-marker sets based on linkage disequilibrium (LD) cutoff using the software Plink v1.9 (Purcell et al., 2007). Different R-squared cutoff values of LD (0.9, 0.85, 0.8, 0.75, 0.7, 0.65, 0.6, 0.55, 0.5, 0.45, 0.4, 0.35, 0.3, 0.25, 0.2, 0.15, and 0.1) were used to generate pruned marker sets, and six marker sets were selected by a number of markers. These marker sets were analyzed for statistical value of LD within 50 kb using Plink v1.9 and were used for cross-validation of five fruit-related traits using the same procedure described above.

### Testing of Genomic Prediction by Population Structure

To estimate the effect of population structure on genomic prediction, the core collection was dissected into its different species. First, the *C. annuum* complex, including *C. annuum*, *C. chinense*, and *C. frutescens* accessions (302 lines), was used to train the models and predict the GEBVs of PD RILs. Second, only *C. annuum* lines (229 lines) were used for training.

With each training population, a combination of three genomic prediction methods (gblupRR, RKHS, and random forest) and two marker sets (18,663 SNPs, 9,282 SNPs) were tested on another population, PD RILs. Selected methods and marker sets were used to train the final genomic prediction model for each trait with whole genotypic datasets. Genotypic datasets of PD RILs were used as test data for the trained model. The prediction accuracies were confirmed by Pearson correlation between GEBVs of the PD RILs and mean of phenotypic values observed for 2 years. Only four traits (fruit length, fruit shape, fruit width, and fruit weight) were tested because data were not available for PD RILs.

## Results

### Phenotypic Variability and Heritability of Fruit-Related Traits

**Figure 1:**
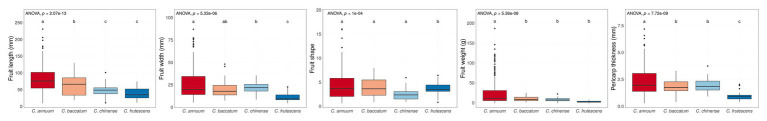
Box plots of four fruit-related traits grouped by species. We investigated the phenotype of the core collection, which contained only one *Capsicum chacoense* accession so, the phenotype of *C. chacoense* was excluded. Boxes indicate the range of upper quartile and lower quartile, and the bar in the box is the median. Whiskers from upper quartile to maximum and lower quartile to minimum are vertical lines. Black spots are outliers. The small alphabets showed the difference among the species. These significances were calculated by Dancan’s least significant range (LSR) test. The core collection has a distinct population structure by species. These phenomena were commonly observed for all four traits.

Phenotype values were highly varied in the training population (*C. annuum* complex). The phenotypic variation ranged from 6.67 to 298.67 mm in fruit length, 0.44 to 43.14 in fruit shape, 3.38 to 101.33 mm in fruit width, 0.09 to 242.50 g in fruit weight, and 0.09 to 10.80 mm in pericarp thickness, respectively (Table 1). For all traits, phenotypic values for *C. annuum* were significantly different from those of the other three species groups (*C. baccatum*, *C. chinense*, and *C. frutescens*), indicating that population structure exists within the core collection (Figure 1).

**Table 1:** Descriptive statistics of raw phenotypic values and broad-sense heritability.

Trait^a^	Minimum	Median	Maximum	Mean ± SD	Heritability
FL (mm)	6.67	66.67	298.67	72.72 ± 40.26	0.975
FS	0.44	3.25	43.14	3.97 ± 17.42	0.987
FWd (mm)	3.38	19.00	101.33	24.80 ± 2.95	0.976
FWg (g)	0.09	8.57	242.50	21.22 ± 32.48	0.969
PT (mm)	0.09	1.80	10.80	2.22 ± 1.48	0.954

a FL, fruit length; FS, fruit shape; FWd, fruit width; FWg, fruit weight; PT, pericarp thickness.

**Figure 2:**
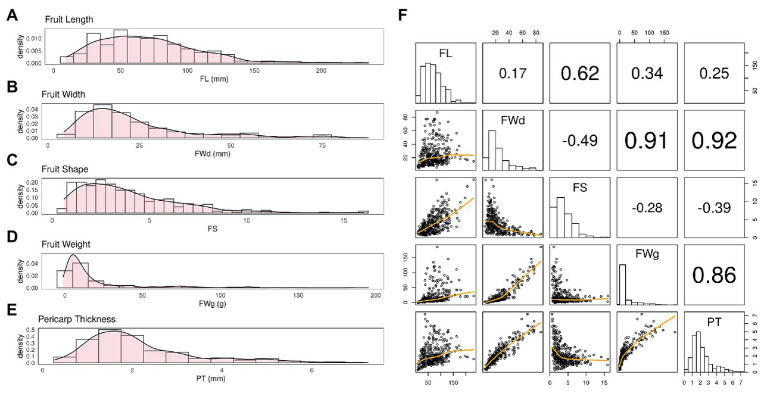
Distribution of BLUP values varies by trait. **(A)** Fruit length (FL) showed a fairly normal distribution with slight skewness. **(B,C)** Fruit width (FWd) and fruit shape (FS) showed a similar distribution to FL. **(D)** Fruit weight (FWg) showed the most skewed distribution among the four traits. **(E)** Pericarp thickness (PT) also showed a fairly normal distribution. **(F)** The correlation was 0.92 for FWd and PT, 0.91 for FWd and FWg, and 0.86 for FWg and PT. These Pearson correlation among traits showed highly correlation among FWd, FWg, and PT.

BLUP values calculated for each fruit-related trait showed varying distributions (Figure 2). BLUP values for all four traits showed a slightly skewed distribution, with the greatest bias in fruit weight. Estimated broad-sense heritability (*H*) of the four traits was similar. All traits showed high heritability (>0.90; Table 1).

Fruit width, fruit weight, and pericarp thickness were highly correlated with Pearson correlation values over 0.85. However, FL showed less correlation with the other traits (Figure 2E).

### SNP Marker Distribution and Linkage Disequilibrium

Genotyping of the core collection and PD RILs identified 16,706,014 common SNPs of which 91,434 remained after prefiltering for SNPs with less than 30% missing alleles. The missing genotypes of the remaining SNPs were phased and imputed using BEAGLE. After imputation, SNPs with minor allele frequency (MAF) less than 5% were removed, giving a total of 18,663 remaining SNPs.

All 18,663 SNPs were pruned based on LD such that the remaining SNPs represented the high LD region. We selected seven marker sets from the total marker set, generated using different LD cutoff values: all SNPs (18,663 SNPs), 2nd set (9,282 SNPs), 3rd set (4,896 SNPs), 4th set (2,578 SNPs), 5th set (1,391 SNPs), and 6th set (711 SNPs). Each pruned marker set showed well-distributed SNPs over 12 chromosomes (Table 2). Decreased SNPs in the centromeric regions of the pepper genome indicated weak LD values (Supplementary Figure 1).

**Table 2:** Marker distribution and linkage disequilibrium (LD) cutoff for each marker set.

Marker set	Number of markers	LD cutoff	Median	Mean ± SD	Maximum
All SNPs	18,663		0.119	0.343 ± 0.392	1.000
2nd set	9,282	0.80	0.027	0.137 ± 0.207	0.800
3rd set	4,896	0.4	0.013	0.067 ± 0.102	0.398
4th set	2,758	0.2	0.005	0.029 ± 0.048	0.196
5th set	1,391	0.1	0.003	0.011 ± 0.020	0.098
6th set	711	0.05	0.001	0.006 ± 0.010	0.044

### Genetic Clustering Analysis of the Core Collection

**Figure 3:**
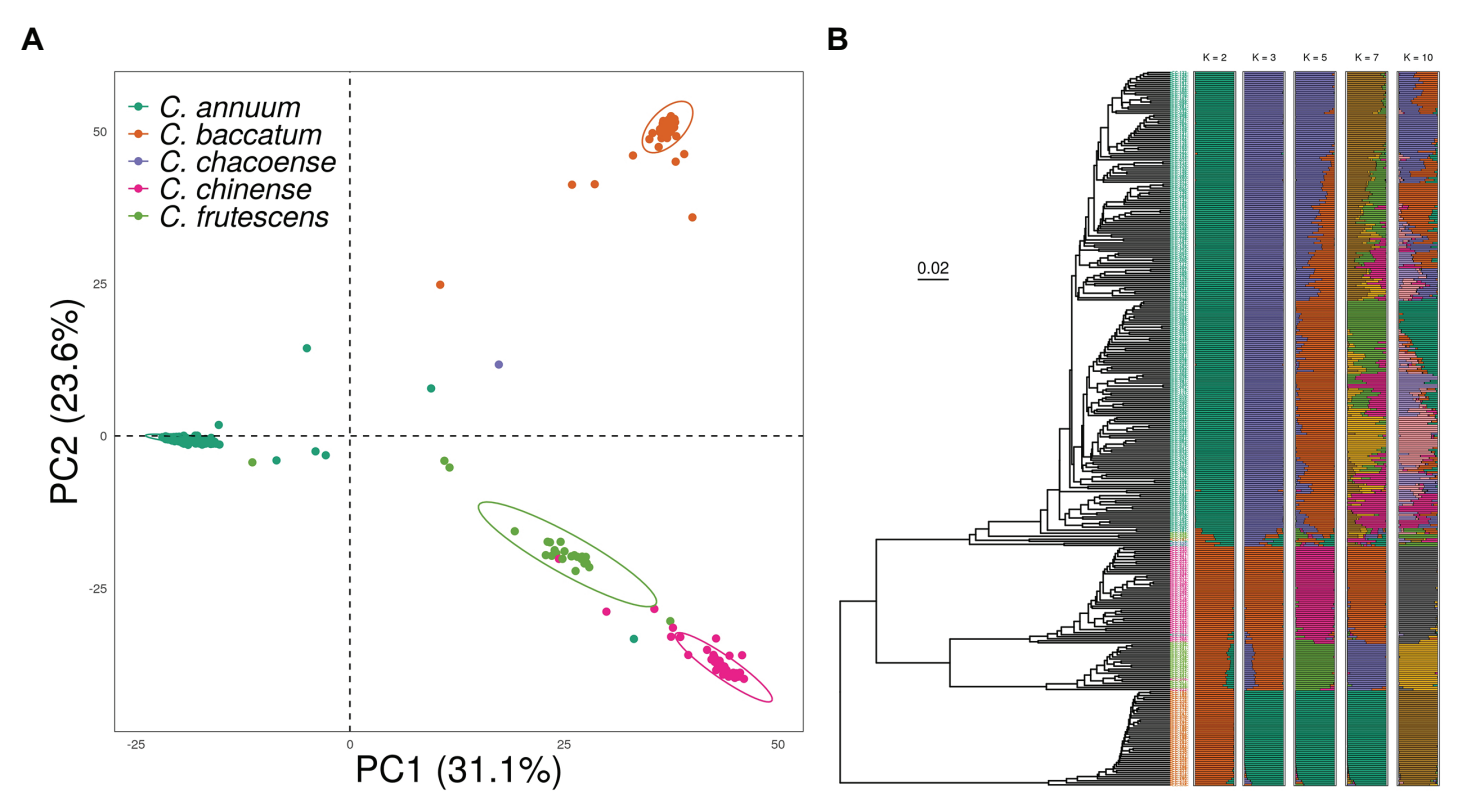
Population structure of the pepper core collection showing a distinct population structure based on species. **(A)** Principal component analysis (PCA) indicates strong separation of each cluster. **(B)** Phylogenetic tree made by using unweighted pair group method with arithmetic mean (UPGMA) showing a similar clustering pattern to PCA. In the ADMIXTURE Q plots, accessions cluster as *C. annuum* or other species when *K* = 2. When *K* = 5, almost all samples cluster into four species, with additional clustering within *C. annuum*.

To investigate the effect of population structure on genomic prediction, we conducted principal component analysis (PCA), phylogenetic tree reconstruction, and admixture analysis using 18,663 SNPs obtained from 350 accessions from the pepper core collection. The pepper core collection showed a significant population structure due to the presence of different species with various origins. Although some of the accessions showed a slight admixture, PCA results showed four genetic clusters according to *Capsicum* species classification (Figure 3A). The first and second principal components explained 31.1 and 23.6% of the variation within the core collection, respectively.

Clusters obtained in the phylogenetic tree were similar to those from PCA. There was a clear separation between *C. baccatum* and the other three species. The four-species group was divided into two clusters, of which the major group mainly consisted of *C. annuum* accessions. We observed a clear genetic relationship between *C. chinense* and *C. frutescens*, which are known as the *C. annuum*-complex species. Finally, we conducted an *ADMIXTURE* analysis with the number of ancestral populations (*K*) ranging from 1 to 12. The cross-validation error was nearly saturated from *K* = 7, with a value of 0.3, indicating the high divergence level within the core collection (Supplementary Figure 2). However, with *K* = 5, the core collection clustered well according to species. The additional substructure was found in *C. annuum* accessions by increasing *K*. However, comparing the structure of *K* = 7 and this of *K* = 10, both structures were similar after the structure was clustered by species in *K* = 5 (Figure 3B).

### Evaluation of Genomic Predictions in Pepper

We evaluated GS using the core collection, which showed genetic diversity and population structure. We investigated the effects of various genomic prediction methods and marker density on prediction accuracy using cross-validation. Based on cross-validation results, we then tested genomic prediction across the population under specific conditions.

#### Cross-Validation Results for Different Genomic Prediction Methods

**Figure 4:**
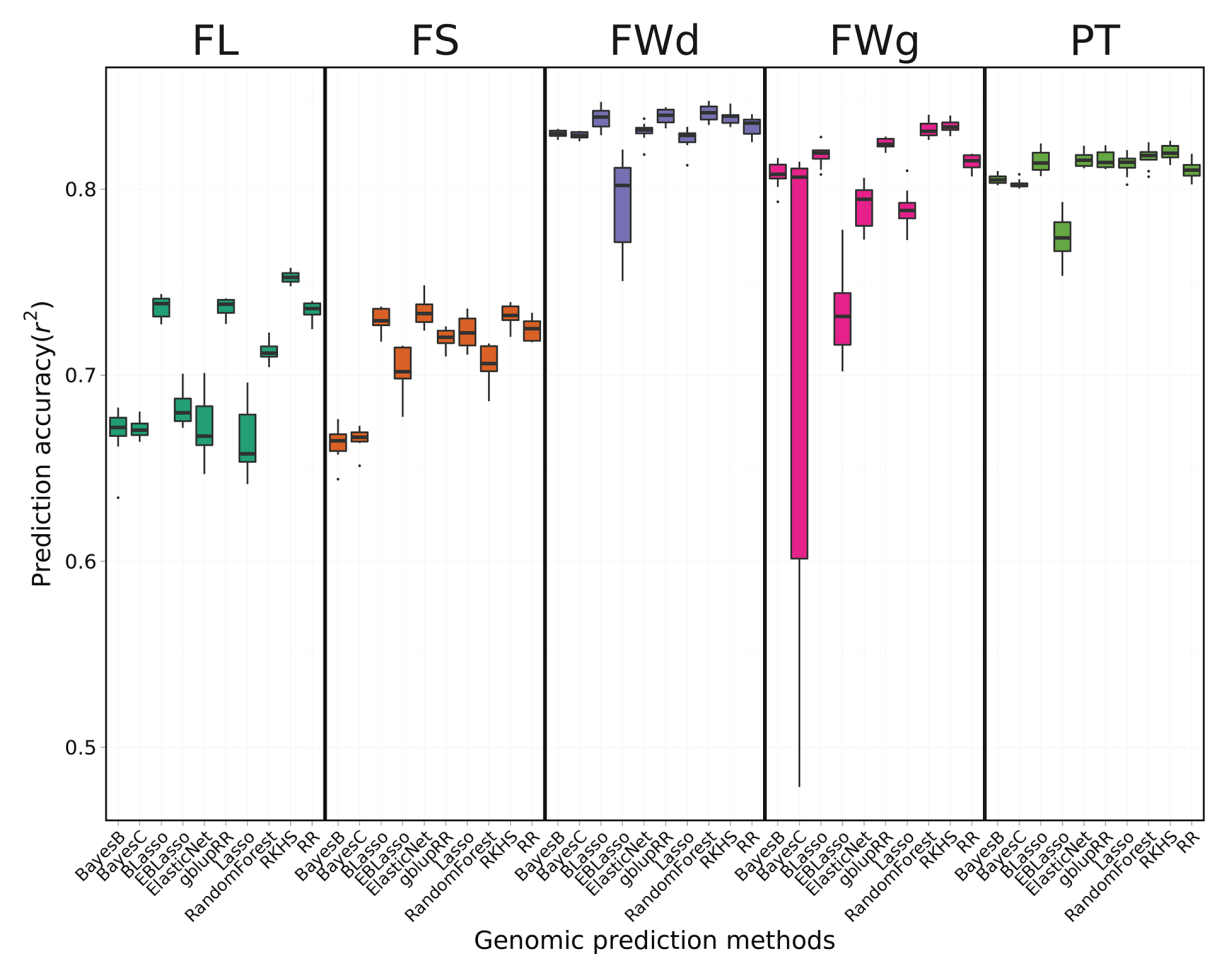
Prediction accuracy of cross-validation across four fruit-related traits by different genomic prediction methods. BLUP values for each trait were applied to 10 different genomic prediction models. Boxes indicate the range of upper quartile and lower quartile, and the bar in the box is the median. Whiskers from upper quartile to maximum and lower quartile to minimum were vertical lines. Black spots were outliers. Reproducing Kernel Hilbert Space (RKHS) was the most effective model for fruit length (FL). Random forest showed high performance for fruit width (FWd). Among the four traits, FWd had the highest accuracy with the highest heritability. Random forest showed the highest accuracy for fruit weight (FWg). Prediction accuracy of the Bayes C method was unstable. Random forest showed the highest performance for pericarp thickness (PT).

Prediction accuracies (Pearson’s correlation) differed for the fruit-related traits evaluated and genomic prediction methods used, ranging from 0.66 to 0.84 (Figure 4). The phenotype prediction accuracy for fruit width was the highest, whereas that for FL was the lowest.

The average prediction accuracy of FL across 10 genomic prediction methods was 0.70, with RKHS having the highest prediction accuracy of 0.75 and Lasso, Bayes B, and Bayes C showing the lowest accuracy of 0.67. The mean of prediction model accuracy was 0.71 in fruit shape. The average prediction accuracy of fruit width across various methods was 0.83, which was higher than the average prediction accuracy of the other traits. The two kernel-type methods (RKHS and gblupRR) and random forest were the most effective genomic prediction methods for fruit width, showing prediction accuracy of 0.84. The mean of prediction accuracy for fruit weight and pericarp thickness were 0.79 and 0.81, respectively. RKHS and random forest resulted in the highest prediction accuracy of 0.84. Bayes C showed highly variable prediction accuracies ranging from 0.48 to 0.83. gblupRR, RKHS, elastic net, and random forest showed similar prediction accuracies for pericarp thickness of approximately 0.82. Among the 10 genomic prediction models, RKHS showed stable prediction accuracies across the four traits (Figure 4).

#### Cross-Validation Results With Different Numbers of Markers

**Figure 5:**
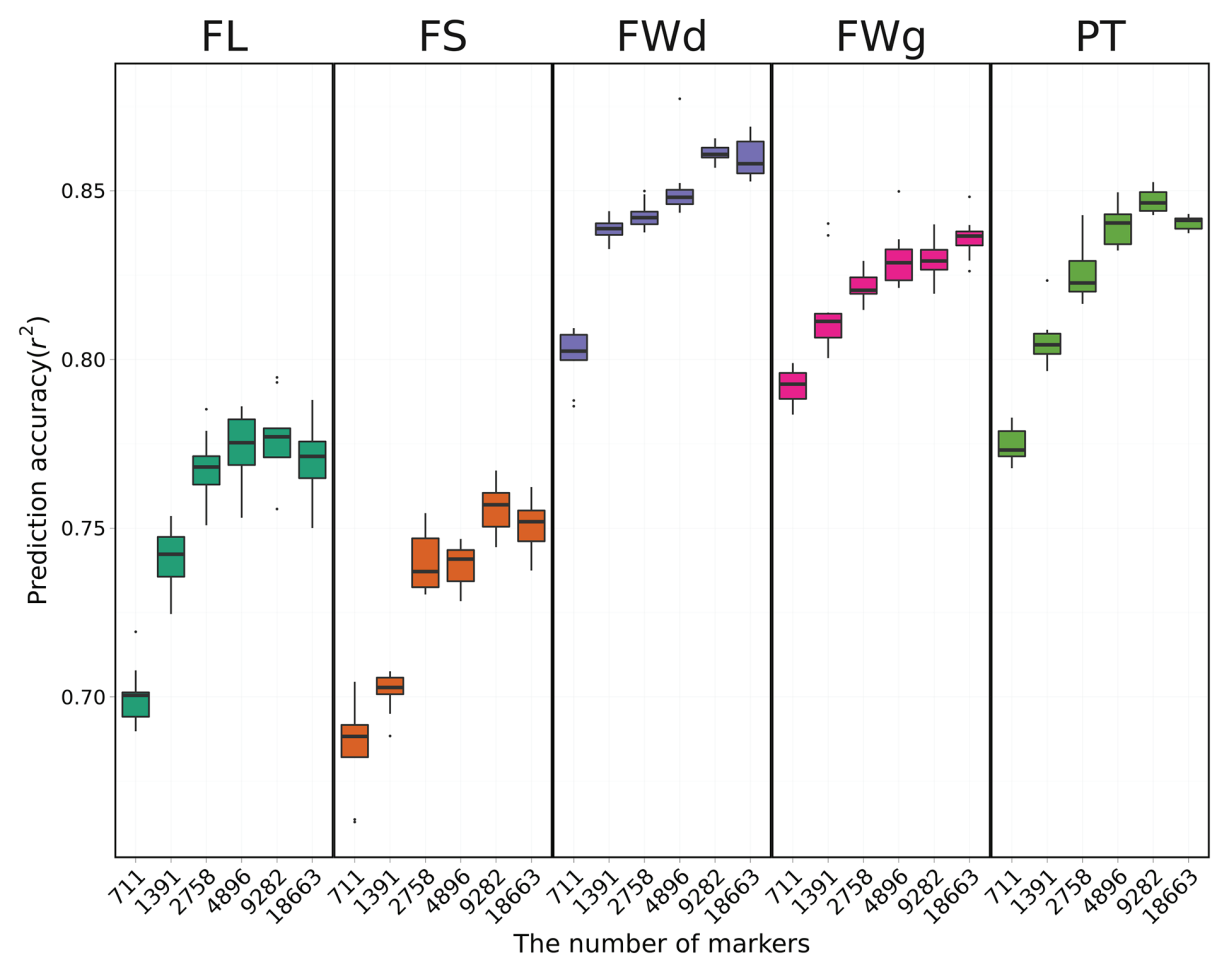
Cross-validation results using the RKHS method with various marker sets. Boxes indicate the range of upper quartile and lower quartile, and the bar in the box is the median. Whiskers from upper quartile to maximum and lower quartile to minimum are vertical lines. Black spots are outliers. Prediction accuracy tends to decrease with fewer markers.

We used only the RKHS model that showed relatively high prediction accuracies to investigate the effect of marker density on genomic prediction. Overall results indicated that prediction accuracy decreased as marker number decreased (Figure 5). Average prediction accuracy was highest for fruit width at 0.83 and lowest for fruit length at 0.73.

Prediction accuracy for fruit length was increased by 10.2% from 0.69 with 431 SNPs to 0.76 with all SNPs (Table 3). However, fruit length was the only trait for which prediction accuracy increased as marker number increased. For the other three traits (fruit width, fruit weight, pericarp thickness), the highest prediction accuracies were obtained for the 3rd set (4,640 SNPs). The highest prediction accuracies for each trait were 0.85 for fruit width, 0.82 for fruit weight, and 0.82 for pericarp thickness. The difference in prediction accuracy between the lowest number of markers and the all-marker set was 4.9, 4.6, and 5.4% for fruit width, fruit weight, and pericarp thickness, respectively.

**Table 3:** Average of prediction accuracy calculated by using the reproducing kernel Hilbert space (RKHS) model with different numbers of markers.

Number of markers	Fruit length		Fruit shape		Fruit width		Fruit weight		Pericarp thickness	
	Avg^a^	SD	Avg	SD	Avg	SD	Avg	SD	Avg	SD
All SNPs (18,663 SNPs)	0.770	±0.010	0.751	±0.007	0.860	±0.005	0.836	±0.006	0.840	±0.002
2nd set (9,282 SNPs)	0.777	±0.011	0.756	±0.007	0.861	±0.002	0.830	±0.006	0.847	±0.003
3rd set (4,896 SNPs)	0.774	±0.010	0.739	±0.006	0.851	±0.009	0.830	±0.008	0.839	±0.005
4th set (2,758 SNPs)	0.768	±0.010	0.740	±0.008	0.843	±0.004	0.821	±0.004	0.826	±0.009
5th set (1,391 SNPs)	0.741	±0.008	0.702	±0.006	0.838	±0.003	0.814	±0.013	0.805	±0.007
6th set (711 SNPs)	0.700	±0.008	0.685	±0.013	0.801	±0.008	0.792	±0.005	0.774	±0.005

a The mean of 10-fold cross-validation values.

### Application of Genomic Prediction Models in a Test Population

For the practical application of a GS method, the GS model should be validated across the population. To test our genomic prediction models, we applied three genomic prediction methods to a different population. The RIL population (PD RILs) was derived from two parental lines included in the pepper core collection. Since pericarp thickness data were not available for the PD RILs, we only investigated fruit length, fruit shape, fruit width, and fruit weight.

When the model was trained with all SNPs set, the RKHS model showed the highest prediction accuracy for fruit shape (0.487), followed by fruit width (0.468), fruit weight (0.430), and fruit length (0.318; Table 4). Overall, the random forest model showed lower prediction accuracies (0.285–0.353) than the other models, but in the case of fruit shape trait, it showed the highest prediction accuracy (0.594). Using the gblupRR model, prediction accuracy of fruit length was the highest (0.352), whereas the rest of accuracies were moderate. Similar to the cross-validation results, fruit width and fruit weight were predicted with greater accuracy than fruit length across the population, but for fruit shape, it showed the highest accuracies in RKHS and random forest dissimilar to the cross-validation results. RKHS showed higher and more stable prediction accuracies than the other genomic prediction methods. However, for fruit length, gblupRR showed slightly higher prediction accuracy than RKHS.

**Table 4:** Results of genomic prediction tests on PD recombinant inbred lines (RILs) by training subset using the all-marker set [18,663 single-nucleotide polymorphisms (SNPs)] and 2rd set (9,282 SNPs).

Subset of core collection	Trait^a^	gblupRR		RKHS		Random forest	
		18,663 SNPs	9,282 SNPs	18,663 SNPs	9,282 SNPs	18,663 SNPs	9,282 SNPs
*Capsicum annuum* complex (302 lines)	FL	0.352	0.317	0.336	0.318	0.285	0.333
	FS	0.443	0.387	0.487	0.397	0.594	0.583
	FWd	0.457	0.484	0.468	0.499	0.320	0.343
	FWg	0.403	0.436	0.430	0.483	0.353	0.351
*C. annuum* only (229 lines)	FL	0.363	0.329	0.345	0.330	0.328	0.277
	FS	0.408	0.387	0.433	0.397	0.498	0.468
	FWd	0.452	0.481	0.436	0.494	0.373	0.407
	FWg	0.401	0.437	0.427	0.480	0.415	0.434
*Capsicum chinense* and *Capsicum frutescens* (73 lines)	FL	−0.308	−0.205	−0.156	−0.196	−0.006	−0.042
	FS	0.245	0.391	−0.008	0.357	−0.190	0.018
	FWd	0.006	0.126	−0.033	0.079	−0.129	−0.127
	FWg	−0.034	0.033	−0.148	0.000	−0.134	−0.094

a FL, fruit length; FS, fruit shape; FWd, fruit width; FWg, fruit weight; PT, pericarp thickness.

**Figure 6:**
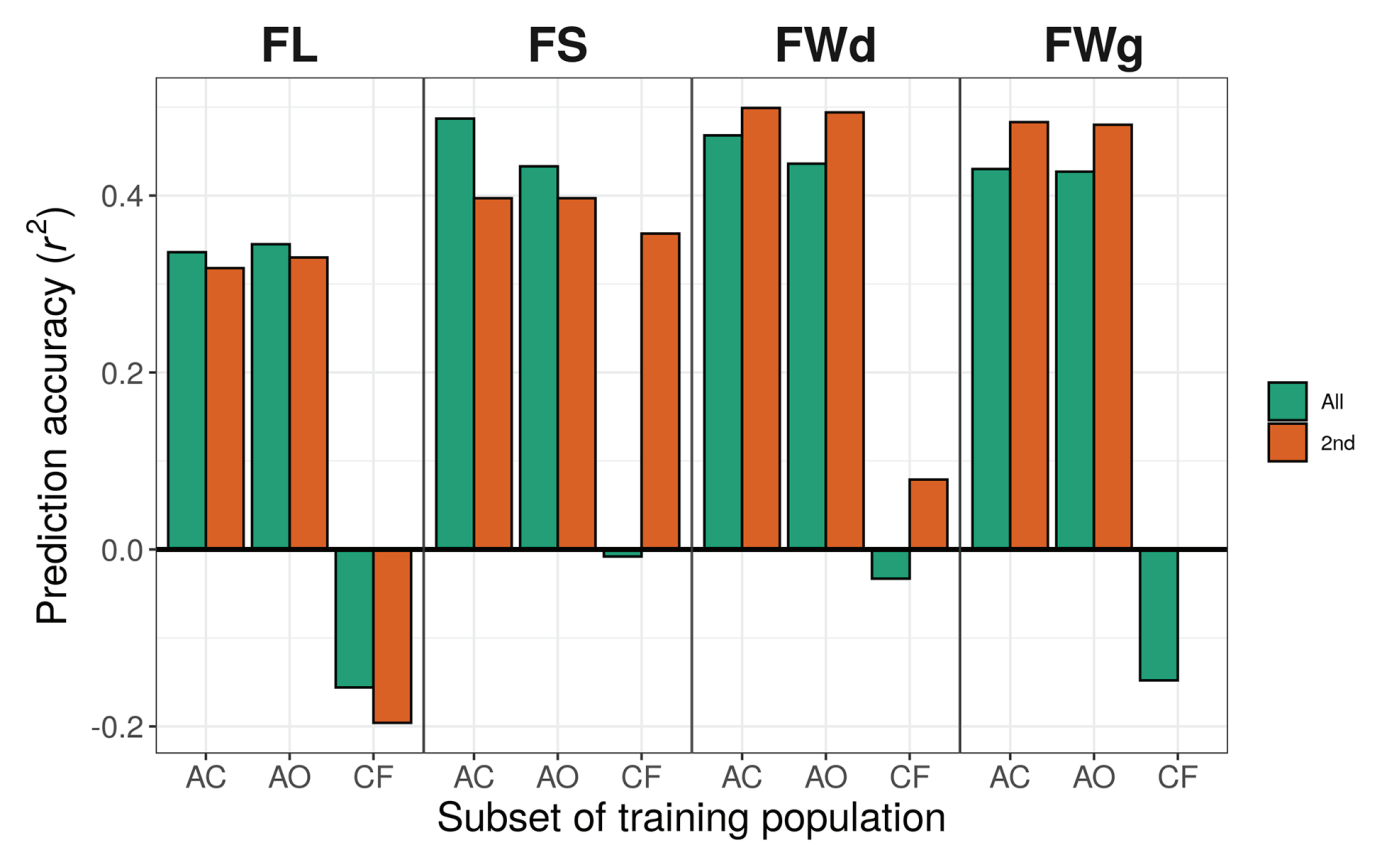
Prediction accuracy using different training populations with RKHS. Green bars indicate results using the all-marker set [18,663 single-nucleotide polymorphisms (SNPs)]; orange bars indicate results using the 2nd marker set (9,282 SNPs). Training populations: AC, *C. annuum* complex (302 lines); AO, *C. annuum* only (229 lines); CF, the rest of the lines except *C. annuum* population (*C. chinense* and *C. frutescens*; 73 lines).

In the case of the all-marker set (18,663 SNPs), when we used only *C. annuum* accessions for training the models, prediction accuracies for fruit length predicted by gblupRR, RKHS, and random forest were slightly increased to 0.363, 0.345, and 0.328, respectively, compared with 0.352, 0.336, and 0.385 using *C. annuum* complex lines. However, when models were trained with the population, which were genetically far from the test population, all models could not predict most traits. In conclusion, the models trained with *C. annuum* complex showed stable prediction accuracies than the case of models trained with only *C. annuum* lines (Figure 6; Table 4).

## Discussion

Pepper fruit-related traits are not only critical determinants for marketable quality but also directly affect its yield and postharvest quality. Therefore, fruit-related traits are one of the most important factors during selection in pepper breeding programs. Here, we investigated the effect of the number of markers, prediction methods, and population structure for prediction of fruit-related traits from genotype data.

### Phenotypic Variation and Population Structure

We calculated the best linear unbiased predictor (BLUP) of each core collection line by fitting a linear mixed effect model for several phenotypic data. BLUP values calculated for each trait were used for further analysis. The BLUP values for five fruit-related traits (fruit length, fruit shape, fruit width, fruit weight, and pericarp thickness) showed a slightly skewed distribution. To investigate the effect of heritability on genomic prediction, we used the variance components from linear mixed effect models to estimate heritability of the traits. Some previous studies have reported high values of heritability in fruit-related traits (Ben-Chaim and Paran, 2000; Naegele et al., 2016). However, it showed way higher values than our expectations indicating some level of overestimation caused by relatively old passions of phenotyping methods and imbalanced experimental design. In a recently reported pepper GWAS study (Colonna et al., 2019), high-resolution phenotyping methods by fruit scanning have been suggested and improved the results detecting novel causal variants. In a future GS study on pepper, improved phenotypic methods could lead to more precise results.

Our analysis grouped the diverse accessions of the pepper core collection into distinct genetic clusters (Lee et al., 2016). These clusters allowed moderate differentiation of fruit-related phenotypic traits. Genetic clustering using *ADMIXTURE* showed similar results to PCA and hierarchical clustering. Although the error was saturated at *K* = 7, the accessions was clustered by species at *K* = 5. When the error was saturated, a strong substructure was not found additionally, and additional structures were clustered in only *C. annuum* accessions. It could be explained that the previous study showed that *C. annuum* accessions were clustered according to its origin (Lee et al., 2016). Similar to previous studies, we obtained clear clustering between the *C. annuum* complex (*C. annuum*, *C. chinense*, and *C. frutescens*) and *C. baccatum* except for some admixed lines. Especially, *C. baccatum* accessions were genetically distinct from the other three species (*C. annuum* complex). There was one *C. chacoense* accession, which was recently reported as *C. baccatum* complex (Carrizo García et al., 2016). However, in the core collection, it did not cluster in the *C. baccatum* cluster. To gain a deeper knowledge of the genetic resources of pepper, precise accession identification methods, adjustment of ambiguous lines in gene banks, and taxonomic studies are needed in the future. In conclusion, to improve reliability and reduce the ambiguous population structure, we excluded the *C. baccatum* and *C. chacoense* in further genomic prediction analysis.

### Prediction Accuracy of Genomic Selection in the Pepper Core Collection

Prediction accuracy varies among genomic prediction methods according to their assumptions and treatments of marker effects (Desta and Ortiz, 2014). Therefore, we investigated the performance of various genomic prediction methods through 10-fold cross-validation. Although there were no significant differences in the prediction accuracy of the methods, gblupRR and RKHS showed consistently high prediction accuracies among the 10 different methods for all traits. The mean of prediction accuracies for fruit length were lower than prediction accuracies for the other traits, highlighting the different genetic architectures of the loci controlling these traits. Highly correlated traits (fruit width, fruit weight, and pericarp thickness) showed similar patterns of prediction accuracy.

Previous studies have shown positive correlation between prediction accuracies of genomic prediction and heritability, and its impacts were relatively larger than other factors (Poland et al., 2012; Desta and Ortiz, 2014). However, we observed no correlation between prediction accuracies and heritability in this study. Although fruit length showed the middle-level heritability (0.971) among the traits tested, its prediction accuracy was lowest. Since all of the fruit-related traits showed high heritability (>0.90), differentiation of prediction accuracies was not clearly detected. Various traits showing a wide range of heritability will need to be tested in future studies to investigate the reliable results of interaction between heritability and prediction accuracy in pepper.

Lower marker density typically resulted in lower prediction accuracy for fruit-related traits, similar to previous studies (Desta and Ortiz, 2014). This can be explained by the fact that a large number of markers can cover all genomic regions (also known as LD block) that correspond to the traits, explaining the majority of marker effects. However, prediction accuracies using the 2nd set with 9,282 SNPs were higher than those using the all-marker set with 18,663 SNPs for fruit length, fruit shape, fruit width, and pericarp thickness, whereas fruit weight had the highest prediction accuracy using the all-marker set. This indicates that the LD levels of loci controlling fruit width, fruit weight, and pericarp thickness may be fully characterized by lower dense marker sets. Therefore, the 2nd marker set could cover all genomic regions controlling the traits, and noninformative SNPs in the all-marker set might decrease prediction accuracy. However, in the case of fruit weight, even the all-marker set could not cover all possible genomic regions, so a denser marker set might improve prediction accuracy.

After selecting a GS model and optimal marker set, we tested our optimized genomic prediction conditions by cross-validation with another population. Across-population genomic prediction was conducted between the core collection and RIL populations. Generally, prediction accuracy among modest numbers of distant panels (across-population) do not enable reliability since two populations share a small proportion of the genome (de los Campos et al., 2015). Although with difficulties in across-population genomic prediction, we obtained moderate-level of prediction accuracy because models were trained with diverse germplasms (Akdemir and Isidro-Sánchez, 2019). The prediction accuracy for fruit length (0.336) was lower than that for fruit shape (0.487), fruit width (0.468), and fruit weight (0.431) according to the result of RKHS models. The difference in prediction accuracy between fruit length and other traits may be caused by the complexity of fruit length or the effect of population structure.

Since GS was introduced, population structure has been a frequent issue for improving prediction accuracy (Spindel and McCouch, 2016), and previous studies have emphasized the use of a genetically linked population as the test population (Desta and Ortiz, 2014). Also, other existing reports suggested that genetic diversity in training population could affect prediction accuracy (de los Campos et al., 2015; Edwards et al., 2019). To improve prediction accuracy, we tested genomic prediction models by comparing different training populations: *C. annuum* complex accessions, *C. annuum* only accessions, and the rest of the accessions except *C. annuum* (*C. chinense* and *C. frutescens*). When the *C. annuum* complex accessions were used as the training population in RKHS method, higher prediction accuracies were obtained in most traits except fruit length. However, in the random forest model, prediction accuracies using the *C. annuum* only accessions were higher for fruit width and fruit weight than those using *C. annuum* complex. The differences in prediction accuracies between two training populations were not so high. However, when only the *C. chinense* and *C. frutescens* accessions were used for training population, prediction accuracies were drastically decreased. This may be due to the genetic distance between the training population and the testing population. Heffner et al. (2011) demonstrated that prediction accuracy above 0.30 would be good enough for applying GS in a winter wheat breeding programs. By obtaining moderate prediction accuracies (>0.30 in all traits) in this study, we showed the potential of GS in pepper breeding with the relatively reasonable costs (small size of training population and low coverage markers).

In this study, we investigated the potential of GS in pepper for fruit-related traits having a high level (>0.90) of heritability. By using the core collection as a training population, with cross-validation, we found effective conditions for GS such as the type of genomic prediction model and the number of markers. The cross-validation results explained about 80% of genetic variation for fruit-related traits in the pepper core collection. We used genomic prediction models trained using a core collection and tested in a different population (RIL population) with narrow genetic diversity and weak population structure. Although the difference in genetic diversity was high between the training population and the test population, we obtained a moderate prediction accuracy. This study provides a simulation of the commercial pepper pre-breeding procedure and deep knowledge of the genetic architecture of pepper fruit-related traits, showing the potential of GS in pepper. This is, to our knowledge, the first genomic prediction study reported in pepper. To improve the prediction accuracy of genomic prediction, integration with larger-scale genomics and phenomics is needed.

## Data Availability Statement

The datasets generated for this study can be found in The National Agricultural Biotechnology Information Center (http://nabic.rda.go.kr/).

## Author Contributions

J-PH and NR contributed equally as the first authors of this work. J-PH conducted the phenotyping in the core collection, modeling of genomic selection, and genomic prediction, while NR conducted the phenotyping in the core collection and PD RILs. H-YL constructed the core collection of pepper and conducted genotyping in the core collection. GK wrote the manuscript and made the figures. J-KK and EY supervised the genomic prediction. B-CK supervised the overall processes and revised the manuscript. J-PH, GK, and B-CK contributed to the article and approved the submitted version.

### Conflict of Interest

The authors declare that the research was conducted in the absence of any commercial or financial relationships that could be construed as a potential conflict of interest.

## Supplementary Material

Supplementary Figure 1 Seven marker sets of the core collection used for cross-validation.

Supplementary Figure 2 Cross-validation error of each *K* in admixture analysis.
