# Supplementary figures and images for: Genomic Selection for Prediction of Fruit-Related Traits in Pepper (Capsicum spp.)
Source: Front Plant Sci. 2020 Oct 28;11:570871. doi: 10.3389/fpls.2020.570871 (PMC7655793; doi:10.3389/fpls.2020.570871)

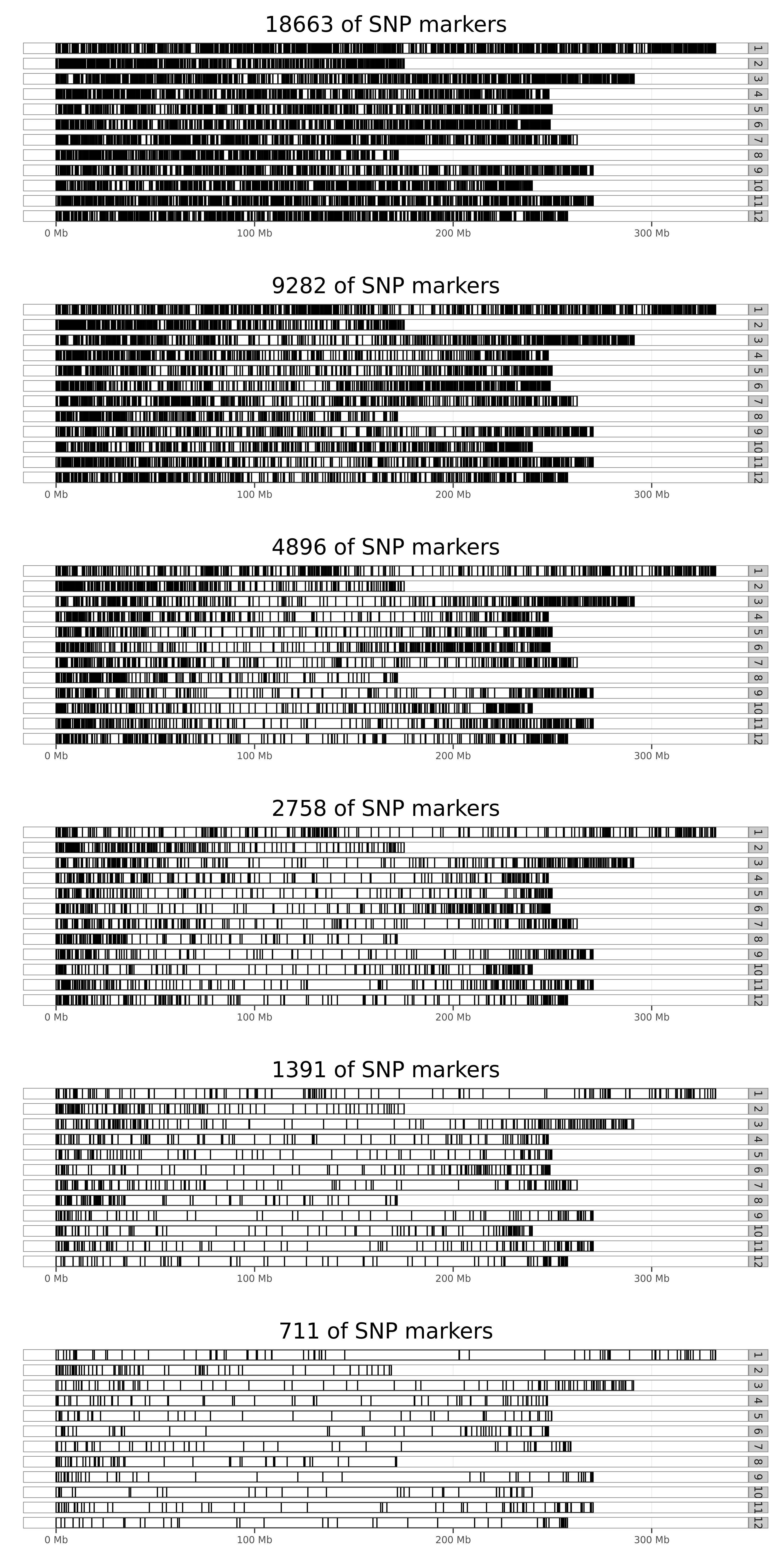

Supplement: Supplementary file 1 [file Image_1.jpeg]

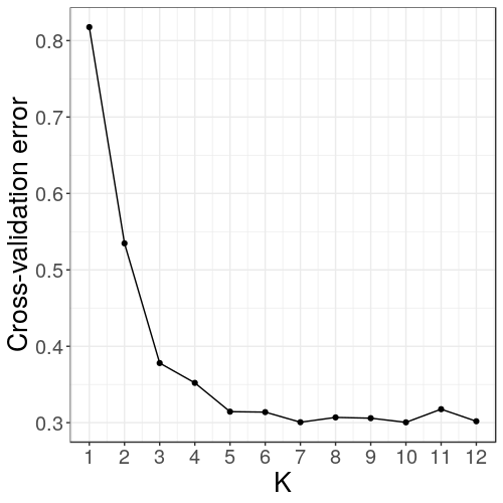

Supplement: Supplementary file 2 [file Image_2.tif]
